# Quality of Life, Coping Strategies, and Psychosocial Support Status of Caregivers Having Children With Neurodevelopmental Disabilities: A Cross-Sectional Study From Bangladesh

**DOI:** 10.7759/cureus.68640

**Published:** 2024-09-04

**Authors:** Anika Tasnim, Marium Salwa, Sharmin Islam, Md Maruf Haque Khan, Mohammad Tanvir Islam, Zubair Ahmed Ratan, Muhammad Ibrahim Ibne Towhid, Mohammad Abdullah Al Mamun, Md. Robed Amin, M Atiqul Haque

**Affiliations:** 1 Department of Public Health and Informatics, Bangabandhu Sheikh Mujib Medical University, Dhaka, BGD; 2 Department of Internal Medicine, Bangabandhu Sheikh Mujib Medical University, Dhaka, BGD; 3 Department of Biomedical Engineering, Khulna University of Engineering and Technology, Khulna, BGD; 4 Department of Epidemiology and Research, National Heart Foundation Hospital and Research Institute, Dhaka, BGD; 5 Department of Internal Medicine, Directorate General of Health Services (DGHS), Dhaka, BGD

**Keywords:** caregiver distress, psychosocial support, stress, quality of life (qol), neurodevelopmental disabilities, bangladesh

## Abstract

Background

Although caregiving is considered a normal phenomenon for parents, delivering care to a child with neurodevelopmental disabilities can be taxing and disastrously impact parents’ quality of life (QoL). This study explored the relationship between QoL, coping strategies, and psychosocial support status of caregivers of children with neurodevelopmental disabilities.

Methodology

This cross-sectional study included 906 caregivers of children having neurodevelopmental disabilities utilizing the World Health Organization Quality of Life Brief and Perceived Stress Scale. A tailored questionnaire gauged coping strategies and psychosocial support. Linear regression was used to identify significant contributors.

Results

Most caregivers (78.8%) experienced a moderate level of stress, and their QoL scores were 14.4 (SD = 2.5) for physical health, 12.0 (SD = 2.4) for psychological health, 14.6 (SD = 1.9) for social relationships, and 12.1 (SD = 2.1) for the environment. Mothers had the lowest QoL of all caregivers. Negative influences on QoL encompassed caregiver and child age, perceived stress, and lower socioeconomic status. A higher coping score positively predicted a high health-related QoL score. Gender differences were observed in psychosocial support sources.

Conclusions

The study underscores the need for policymaking considering findings to develop psychosocial intervention programs for enhancing the QoL of caregivers of children with neurodevelopmental disabilities.

## Introduction

Neurodevelopmental disorders are clinical conditions characterized by early impairments in neurobiological development, resulting in deficiencies in social, individual, professional, or educational functioning. Neurodevelopmental disorders encompass autism spectrum disorders (ASDs), intellectual disabilities, epilepsy, attention-deficit hyperactivity disorder (ADHD), cerebral palsy, etc. [1]. Children with neurodevelopmental disabilities require long-term care from their parents, and delivering such supervision for an extended period can negatively impact the caregiver’s physical and psychological health [2].

Raising a child with neurodevelopmental disabilities can significantly increase parental stress and lead to exhaustion due to the heightened demands of caregiving. Unlike raising a typically developing child, parents of children with neurodevelopmental disabilities often face greater challenges that require enhanced social, psychological, physical, and financial support. According to Belsky’s parental stress model, parenting stress is influenced by multiple interconnected factors, including the characteristics of the child, the parents’ personal resources, family dynamics, and environmental conditions [3]. Children with neurodevelopmental disabilities may exhibit behaviors or have needs that are more intense and unpredictable, contributing directly to increased parental stress. Furthermore, the lack of understanding and support from society can exacerbate feelings of isolation and helplessness, adding to the psychological burden on parents. Financial stress also plays a significant role, as many parents must bear the high costs of specialized care, treatments, and educational resources, often without adequate insurance coverage or social support. These factors are often compounded by a lack of respite care and support networks, which can leave parents feeling overwhelmed and isolated. When family and environmental support systems are lacking or inadequate, the stress experienced by parents can further intensify, negatively impacting their overall well-being and the family’s quality of life (QoL). This interconnectedness of stress factors illustrates how parenting a child with neurodevelopmental disabilities often requires more extensive resources and support compared to raising a typically developing child.

Parents and caregivers of children with neurodevelopmental disabilities often devote the majority of their time to caregiving, limiting their social life and severely impacting their QoL. QoL encompasses various elements such as health, happiness, employment, education, social and intellectual attainments, freedom of action, and freedom of expression. The QoL of caregivers is influenced by a complex interplay of socioeconomic position, parent-child traits, and social support [4].

Neurodevelopmental disabilities pose a significant public health concern in low- and middle-income countries, but the scarcity of robust epidemiological data may underestimate the actual burden. In Bangladesh, over 2.8 million people were identified as suffering from neurodevelopmental disabilities in a recent survey [5]. Research conducted in Bangladesh has revealed an inverse association between the number of children diagnosed with autism and the QoL of their parents. Furthermore, parents of children with autism in Bangladesh experience considerable stress and have diminished QoL [6].

The implementation of coping strategies is vital for safeguarding the physical and mental health of an individual in the face of challenging situations [7]. Coping entails a cognitive reevaluation of the situation to effectively manage it. Adapting positive coping strategies can reduce stress [8]. Studies have shown that parents can better cope with the situation of having a child with neurodevelopmental disabilities if they have support from family or friends, healthcare workers, knowledge of treatment facilities, good relations with other parents, and time for hobbies and religious activities, which can improve their self-efficacy and self-confidence.

However, empirical research is scarce on the QoL and coping strategies of parents of children with neurodevelopmental disabilities in Bangladesh. Therefore, this study explores the relationship between QoL, coping strategies, and psychosocial support status among parents of children with various neurodevelopmental disabilities.

## Materials and methods

Study design and settings

This cross-sectional study enrolled 906 caregivers of children having neurodevelopmental disabilities from eight administrative divisions and 12 city corporation areas of Bangladesh from January to June 2019. In this study, we included caregivers of children with ASD, ADHD, intellectual disability, communication disorders, specific learning disorders, motor disorders, cerebral palsy, Down syndrome, etc. Neurodevelopmental disabilities were collected from the register books of Shishu Bikash Kendra (SBK) in Medical College Hospitals in eight divisions of Bangladesh. The sample size for the study was detected using the mean and standard deviations for each of the four QoL areas discussed in a prior study [9]. Basic information on children with neurodevelopmental disabilities and their contact addresses, including phone numbers, were obtained from the register books. A simple random sampling method was employed to identify caregivers for the study using the registry numbers of each child with neurodevelopmental disability as the sample frame. Data were collected by 15 college graduates with a social sciences background who were trained for the study. The detailed data collection procedure was previously described in a paper [9].

Data collection tool

Measurement of Perceived Stress

The Perceived Stress Scale-10 (PSS-10) is a widely used measure of global perceived stress, which assesses the unpredictability, uncontrollability, and overburdening of an individual’s life in general. The scale consists of 10 items, six of which are positively phrased, and four are negatively phrased. Each item is scored on a response scale ranging from 1 to 4. The total score ranges from 0 to 40. Sample items of the PSS-10 include “In the last month, how often have you been upset because of something that happened unexpectedly?” and “In the last month, how often have you found that you could not cope with all the things that you had to do?” The Bengali version of the PSS-10 is available for free use from Sheldon Cohen’s laboratory [10]. Parental stress scores are categorized into the following three levels: low (0-13), moderate (14-26), and high (27-40). The Cronbach’s alpha of this scale is 0.725.

Measurement of Health-Related Quality of Life

This study used the World Health Organization Quality of Life Scale-Brief (WHOQOL-BREF), a well-validated and appropriate tool for collecting data in cross-cultural settings [11]. The scale consists of 26 items, including two items each from the categories of general health and overall QoL. The remaining 24 items are related to satisfaction and are grouped into the following four categories: physical (seven items), psychological (six items), social relationships (three items), and environmental (eight items). The physical domain of QoL focuses on physical pain, sleep, treatment facilities, dealing with daily life, and physical activities. The psychological domain assesses negative feelings such as anxiety and depression, level of satisfaction, acceptance of bodily appearance, level of concentration, and meaningfulness of life. The social relationships domain examines the individual’s level of containment in relationships and support systems, which may be affected by the child’s disability. The environmental health domain of QoL is concerned with physical safety and security, the home environment, health, and social care. The participants were asked to rate “how much,” “how completely,” “how often,” “how good,” or “how satisfied” they felt for each item in the last two weeks on a five-point Likert scale. Each item of the WHOQOL-BREF was scored from 1 to 5 on a response scale. Three methods were used to calculate the WHOQOL-BREF domain scores, namely, summation of the raw scores of the constituent items, a transformation of the raw scores into scores ranging from 4-20, and a conversion of the 4-20 scores onto a 0-100% scale. Written permission was obtained to use the validated Bengali version of WHOQOL-BREF and the analysis guideline from the World Health Organization.

Measurement of Coping Mechanism and Coping Status for Parenting Stress

Based on a relevant literature review, we developed a tool to measure the coping mechanisms and strategies employed by parents of children with neurodevelopmental disabilities. We summarized key points from various widely accepted assessment tools designed for coping strategies [8,12]. The questionnaire developed for the study comprises 15 items, and participants were requested to rate each item on a five-point Likert scale, with options ranging from “never” (1), “sometimes” (2), “often” (3), “moderate” (4), to “always” (5). These ratings were used to assess the coping strategies employed by individuals in response to different stressful situations. The total score of the questionnaire ranges from 0 to 75. We conducted a thorough validation process for our questionnaire, evaluating its reliability by calculating Cronbach’s alpha, which was found to be 0.7, indicating acceptable reliability. In addition to this, we ensured the questionnaire’s validity through content validity, face validity, and cultural validation. Content validity was assessed by reviewing the questionnaire items against existing literature and theoretical frameworks and consulting experts to ensure the questions adequately represented the domain of interest and covered all necessary aspects without including irrelevant content. Face validity was evaluated by having experts and potential respondents review the questionnaire to ensure that the items were clear, relevant, and straightforward, making the instrument appear credible and appropriate. For cultural validation, we reviewed the questionnaire to identify and address any cultural biases, ensuring it is valid and appropriate across diverse cultural contexts. This comprehensive validation process ensures that the questionnaire is not only reliable but also comprehensive, understandable, and culturally appropriate, enhancing the credibility and generalizability of the data collected.

Measurement of Psychosocial Support Status

A questionnaire was created to evaluate the psychosocial support received by parents of children with neurodevelopmental disabilities. The questionnaire comprises 11 open-ended items, where parents were asked to identify their top three sources of support that help them relax the most. These sources of support may include spouses, involvement in religion, family, friends, entertainment, other children, leisure activities, involvement with health professionals, domestic help, and others. The questionnaire was adapted from relevant literature reviews and customized to align with the specific sociocultural context of the study in Bangladesh.

Sociodemographic and Disease Profiles of the Respondents and Children

A sociodemographic questionnaire was used to collect information on participants’ age, sex, level of education, marital status, employment status, and socioeconomic status (SES). Additionally, details of the children were collected, including their sex, age, diagnosis, birth order, the number of neurodevelopmental disabilities they had, and any other neurodevelopmental diseases that affected their other children.

Statistical analysis

The relationship among sociodemographic characteristics, parenting stress, and QoL of the participants was assessed using independent t-tests and one-way analysis of variance. Additionally, a chi-square test was performed to analyze the relationship between categorical variables. Multiple linear regression analysis was conducted to determine to what extent independent variables explained the outcome variable and which variables were the most significant risk factors. The caregiver’s sex, occupational status, educational status, family type, stress level, coping strategies, and the children’s age, sex, and birth order were considered independent variables. In contrast, QoL was regarded as the outcome variable. All explanatory variables showing a significant association (p < 0.05) with dependent variables in the univariate analysis were included in the multiple regression model. The data were analyzed using SPSS version 26 for Windows (IBM Corp., Armonk, NY, USA), and a p-value of less than 0.05 was considered the level of statistical significance.

Ethical approval

This study received ethical clearance from Bangabandhu Sheikh Mujib Medical University’s Institutional Review Board, under memo number BSMMU/2019/6025. Recognizing the confidentiality of patient data in Bangladesh’s government hospitals, permissions were secured from both the Directorate General of Health Services and hospital directors for access to these records. An informed consent form was developed and provided to each participant before starting data collection. This form included detailed information about the purpose and objectives of the study, the study procedure, the potential benefits and risks of participation, and the identity of the lead investigator. It ensured that participants were fully aware of what their participation entailed and provided their consent voluntarily. To maintain confidentiality and privacy, each participant was assigned a unique identification number, and all data collected were coded to remove any personally identifiable information. The data were securely stored and only accessible to authorized research staff involved in the study. Access to the data was restricted and controlled to ensure that it was used solely for research purposes, preventing unauthorized access or breaches of confidentiality. Participants were informed of their right to refuse participation or withdraw from the study at any time without any consequences, ensuring respect for their autonomy and decision-making. Furthermore, while participants did not receive compensation for their time, this was clearly communicated to them before they agreed to participate in the study, ensuring transparency and setting appropriate expectations.

## Results

Of 906 caregivers, 64% were mothers, 32% were fathers, and 4% were other family members. On average, the caregivers were 35.1 years old (SD = 9.2). Among the children with neurodevelopmental disabilities, 60.5% were male, and 39.5% were female, with an average age of 1.4 years (SD = 0.5).

The majority of caregivers (78.8%) experienced a moderate level of stress, and their QoL scores were 14.4 (SD = 2.5) for physical health, 12.0 (SD = 2.4) for psychological health, 14.6 (SD = 1.9) for social relationships, and 12.1 (SD = 2.1) for the environment. Social relationships scored higher, while psychological health scored lower for most respondents.

According to the findings presented in Table 1, male caregivers, individuals with higher SES, and those with higher secondary education had better health-related QoL (HRQoL). Caregivers who were engaged in any service also reported higher QoL except in the environmental domain. Moreover, caregivers who had male children with neurodevelopmental disabilities were first-time parents of children with neurodevelopmental disabilities and did not have any other children affected by neurodevelopmental disabilities reported a higher QoL. Participants with higher total coping scores showed better social relationship-related QoL. In addition, individuals who received psychosocial support from friends, family, and other sources reported higher QoL related to social relationships. Furthermore, those who received psychosocial support from entertainment sources reported improved QoL related to the environment.

**Table 1:** Distribution of health-related quality of life with different sociodemographic characteristics of the respondents (n = 906). ^#^: Others included caregivers other than parents such as uncle, aunt, grandparents, etc. ^##^: Others included widowed, divorced, separated, etc. The nuclear family included the father, mother, and children. The extended family included grandparents, parents, and children. ^$^: Entertainment sources (e.g., television, radio, mobile, paper, film, leisure activities such as traveling, gardening, walking, running, swimming, etc.). ^$$^: Other helping sources included domestic helpers, health workers, special children’s organizations, etc.

Sociodemographic characteristics	Physical, mean ± SD	Psychological, mean ± SD	Social relationships, mean ± SD	Environmental, mean ± SD
Caregiver’s sex				
Male	14.9 ± 2.3	12.5 ± 2.4	14.7 ± 1.9	11.9 ± 2.1
Female	14.1 ± 2.6	11.9 ± 2.3	14.5 ± 2.0	12.2 ± 2.1
Type of caregivers				
Father	14.9 ± 2.3	12.5 ± 2.5	14.7 ± 2.0	11.9 ± 2.1
Mother	14.2 ± 2.5	11.9 ± 2.3	14.6 ± 1.9	12.3 ± 2.1
Others^#^	12.9 ± 3.2	11.1± 2.7	13.7 ± 1.9	11.7 ± 2.1
Socioeconomic status				
Lower	13.5 ± 2.9	10.9 ± 2.4	14.1 ± 2.2	10.9 ± 2.0
Middle	14.7 ± 2.2	12.4 ± 2.1	14.6 ± 1.8	12.3 ± 1.9
Upper	15.0 ± 2.1	12.9 ± 2.2	15.0 ± 1.9	13.2 ± 1.8
Marital status				
Married	14.4 ± 2.5	12.1 ± 2.4	14.6 ± 1.9	12.1± 2.1
Others^##^	13.6 ± 2.9	11.2 ± 2.5	13.1 ± 2.5	11.8 ± 2.1
Educational status				
Up to Secondary	14.1 ± 2.6	11.7 ± 2.3	14.4 ± 2.0	11.7 ± 2.1
Higher secondary and above	15.2 ± 2.0	13.1 ± 2.1	14.9 ± 1.8	13.1 ± 1.9
Type of family				
Nuclear	14.4 ± 2.5	12.1 ± 2.4	14.6 ± 1.9	12.2 ± 2.1
Extended	14.4 ± 2.6	11.9 ± 2.4	14.5 ± 2.0	12.1 ± 2.1
Occupational status				
Household work	14.1 ± 2.6	11.9 ± 2.3	14.5 ± 1.9	12.2 ± 2.0
Service holders	15.2 ± 1.8	12.9 ± 2.1	14.8 ± 1.9	12.8 ± 2.1
Business	15.1 ± 2.2	12.6 ± 2.6	14.9 ± 1.9	14.3 ± 1.9
Others	13.9 ± 2.8	11.4 ± 2.5	14.2 ± 2.1	10.9 ± 2.1
Children’s sex				
Male	14.5 ± 2.5	12.3 ± 2.4	14.6 ± 1.9	12.3 ± 2.0
Female	14.2 ± 2.5	11.8 ± 2.4	14.5 ± 2.0	11.9 ± 2.2
Birth order of the child				
First	14.7 ± 2.4	12.3 ± 2.3	14.8 ± 1.9	12.3 ± 2.1
Second and above	14.1 ± 2.6	11.8 ± 2.5	14.4 ± 2.1	11.9 ± 2.2
Child’s age at diagnosis of neurodevelopmental disabilities				
≤ 2 years	14.3 ± 2.7	12.1 ± 2.4	14.6 ± 2.1	12.3 ± 2.1
>2 year	14.4 ± 2.4	12.0 ± 2.4	14.6 ± 1.9	12.0 ± 2.1
Having other children with neurodevelopmental disabilities				
No	14.4 ± 2.5	12.1 ± 2.3	14.6 ± 1.9	12.2 ± 2.1
Yes	13.7 ± 2.8	10.9 ± 2.7	13.8 ± 2.3	11.5 ± 2.3
Perceived stress score				
Low	16.1 ± 1.6	14.2 ± 1.8	15.6 ± 1.6	13.6 ± 1.7
Moderate	14.3 ± 2.5	11.9 ± 2.2	14.5 ± 1.9	11.9 ± 2.0
High	13.5 ± 2.7	11.1 ± 2.7	13.9 ± 2.3	12.0 ± 2.4
Total coping score	14.4 ± 2.5	12.1 ± 2.4	14.6 ± 1.9	12.1 ± 2.1
Psychosocial support status				
From friends and family	14.4 ± 2.6	11.9 ± 2.5	14.5 ± 2.1	12.1 ± 2.1
From entertainment^$^	14.8 ± 2.5	12.6 ± 2.2	14.5 ± 2.0	12.9 ± 2.0
From other helping sources^$$^	13.5 ± 2.4	10.9 ± 2.3	14.1 ± 2.3	11.1 ± 2.2

**Figure 1:**
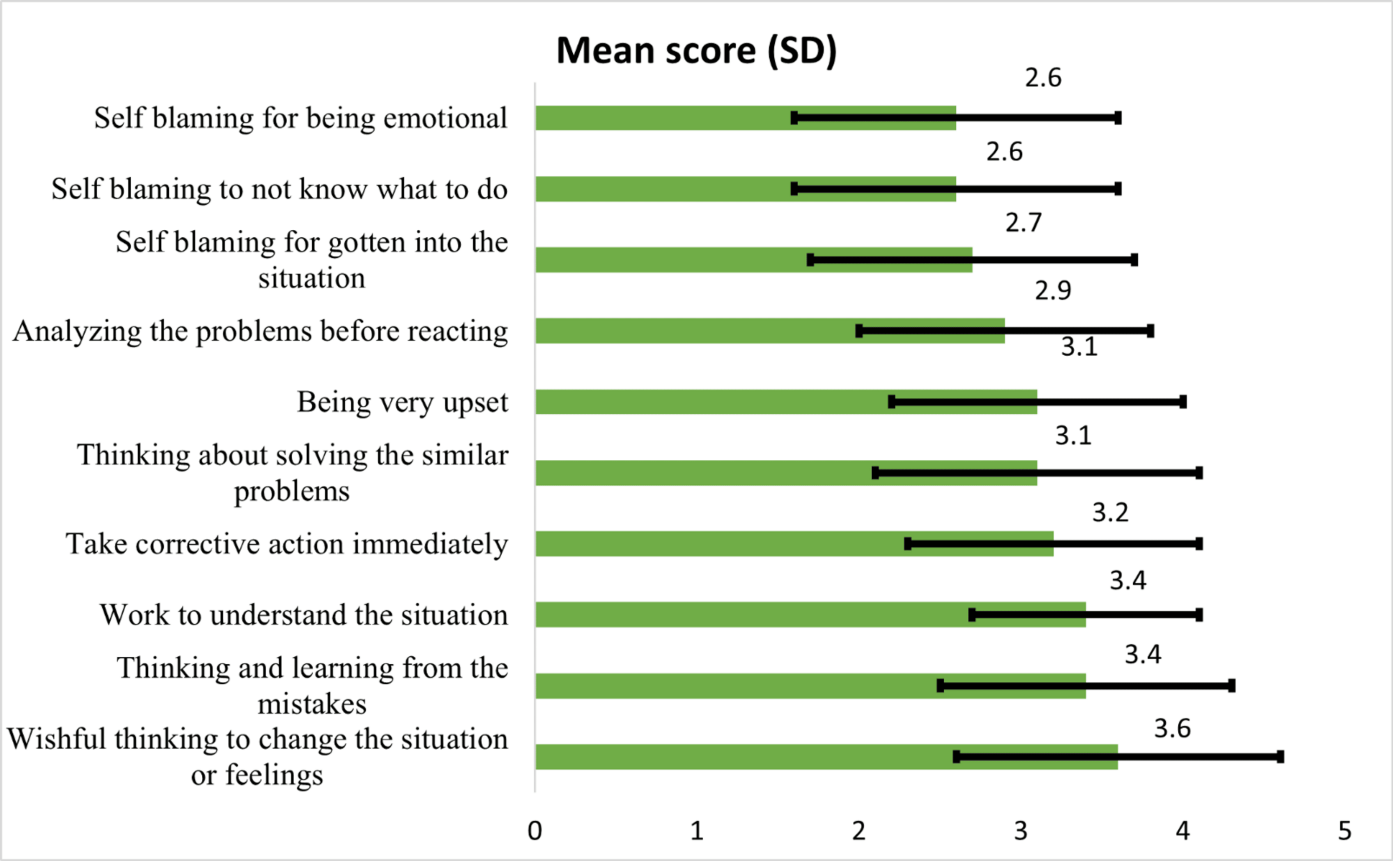
Most frequently used coping strategies among respondents (n = 906).

Figure 1 illustrates the various methods individuals utilized to manage stress or difficulties. These methods ranged from wishful thinking for situational or emotional change, reflecting on and learning from mistakes, understanding the situation deeply, immediate action taking, solving similar past problems, experiencing intense emotions, analyzing problems before responding, and various forms of self-blame related to the situation, decision-making, or emotional responses.

Table 2 reveals that 74% of participants reported receiving psychosocial support from partners, family, friends, and other children. Among them, 77.6% of respondents received support from their spouses, 36% from families, 28.9% from friends, and 14% from other children. Entertainment sources were helpful for 25% of caregivers, and 7% received support from healthcare providers, domestic help, and organizations.

**Table 2:** Distribution of the psychosocial support status of the respondents (n = 906). The chi-square test was used to compare the proportions of stress facilitators between male and female respondents. *: Significant at 0.05 level.

Rank	Facilitator of stress	Frequency (%)			
		Total (n = 906)	Male (n = 301)	Female (n = 605)	P-value
1	Spouse	703 (77.6)	233 (77.4)	470 (77.7)	0.933
2	Religious communities and activities	541 (59.7)	181 (60.1)	360 (59.5)	0.886
3	Family	327 (36.1)	93 (30.9)	234 (38.7)	0.023^*^
4	Friends	262 (28.9)	106 (35.2)	156 (25.8)	0.004^*^
5	Entertainment	137 (15.1)	41 (13.6)	96 (15.9)	0.431
6	Other children	127 (14.0)	28 (9.3)	99 (16.4)	0.004^*^
7	Leisure activities	116 (12.8)	47 (15.6)	69 (11.4)	0.091
8	Domestic helpers	30 (3.3)	4 (1.3)	26 (4.3)	0.018^*^
9	Health worker	21 (2.3)	10 (3.3)	11 (1.8)	0.165
10	Special children’s organization	12 (1.3)	7 (2.3)	5 (0.8)	0.118

Notably, female caregivers received significantly more psychosocial support from family (p = 0.023), other children (p = 0.004), and domestic workers (p = 0.018) compared to male caregivers. On the other hand, male caregivers received significantly more psychosocial support from friends (p = 0.004) compared to their female counterparts.

The findings from the multiple linear regression analysis, as presented in Table 3, revealed that several variables, including the age of the respondents, PSS score, age of the child, caregivers’ employment status, and SES, had a significant impact on HRQoL in the physical health domain. The study revealed that the higher age of the respondents (B = -0.060, p < 0.001), higher PSS score (B = -0.114, p < 0.001), higher age of the indexed child (B = -0.071, p < 0.001), and unemployment status (relative to employment) (B = -0.598, p < 0.05) were significant negative predictors of HRQoL in the physical health domain. Furthermore, middle and upper SES (relative to lower SES) (B = 1.02, p < 0.001) were significant positive predictors of HRQoL in physical health.

**Table 3:** Summary of a multiple regression analysis of the physical, psychological, social relationships, and environmental health in WHOQOL-BREF. The rows are the independent variables and the columns are the dependent variables. B: unstandardized partial regression coefficient; significance level: ** p < 0.05, *** p <0.01. #: Others included fathers and other caregivers. $: Entertainment sources (e.g., television, radio, mobile, paper, film, leisure activities such as traveling, gardening, walking, running, swimming, etc.). ##: Other helping sources included domestic helpers, health workers, special children’s organizations, etc. N.B.: No multicollinearity among variables was observed, and standard P-P plots of regression standardized residuals of the dependent variable were acceptable. SE: standard error; SES: socioeconomic status; PSS: Perceived Stress Scale; WHOQOL-BREF: World Health Organization Quality of Life Scale-Brief

Variables	Physical	Psychological	Social relationships	Environmental
	B (95% CI)	B (95% CI)	B (95% CI)	B (95% CI)
Age of respondent (years)	-0.060 (-0.083 to -0.037)***	-0.029 (-0.049 to 0.008)***	-0.010 (-0.030 to 0.010)	-0.009 (-0.028 to 0.010)
Type of caregivers				
Mother (ref. others^#^)	-0.271 (-0.787 to 0.245)	-0.245 (-0.705 to 0.215)	0.286 (-0.155 to 0.727)	0.431 (0.006 to 0.856)**
Educational level				
Higher secondary and above (ref. up to secondary)	0.324 (-0.045 to 0.693)	0.410 (0.082 to 0.739)**	0.084 (-0.231 to 0.400)	0.693 (0.389 to 0.997)***
SES				
Middle and upper (ref. lower)	1.02 (0.686 to 1.358)***	1.083 (0.784 to 1.383)***	0.541 (0.254 to 0.829)***	1.320 (1.043 to 1.597)***
PSS score	-0.114 (-0.144 to -0.084)***	-0.153 (-0.180 to -0.126)***	-0.089 (-0.114 to -0.063)***	-0.069 (-0.094 to -0.044)***
Age of child	-0.071 (-0.115 to -0.028)***	-0.098 (-0.137 to -0.059)***	-0.021 (-0.058 to 0.017)	-0.055 (-0.091 to -0.019)***
Sex of the child				
Male (ref. female)	0.058 (-0.241 to 0.357)	0.172 (-0.095 to 0.439)	0.010 (-0.246 to 0.265)	0.080 (-0.166 to 0.327)
Birth order of the child				
First order (ref. second and above)	0.134 (-0.194 to 0.461)	0.270 (-0.022 to 0.562)	0.331 (0.051 to 0.611)**	0.197 (-0.073 to 0.467)
Occupational status				
Unemployed (ref. employed)	-0.598 (-1.065 to -0.132)**	-0.013 (-0.429 to 0.403)	-0.228 (-0.627 to 0.171)	0.188 (-0.196 to 0.572)
Marital status				
Yes (ref. unmarried)	0.433 (-0.390 to 1.256)	0.482 (-0.252 to 1.215)	1.291 (0.587 to 1.995)***	0.296 (-0.383 to 0.974)
Having children affected with neurodevelopmental disabilities				
Yes (ref. no)	-0.177 (-0.786 to 0.433)	-0.596 (-1.139 to -0.053)**	-0.518 (-1.04 to 0.003)**	-0.134 (-0.636 to 0.367)
Number of neurodevelopmental disabilities				
> 1 (ref. 1)	-0.048 (-0.343 to 0.246)	0.003 (-0.259 to 0.266)	0.026 (-0.226 to 0.277)	-0.018 (0.260 to 0.225)
Family type				
Nuclear (ref. extended)	0.102 (-0.201 to 0.405)	0.226 (0.044 to 0.496)	0.087 (-0.172 to 0.346)	0.098 (-0.151 to 0.348)
Time at diagnosis				
≤2 years (ref. >2 years)	-0.065 (-0.387 to 0.257)	-0.337 (-0.624 to -0.050)**	-0.048 (-0.323 to 0.228)	-0.243 (-0.509 to 0.022)
Total coping score	-0.002 (-0.027 to 0.024)	0.023 (0.000 to 0.046)**	0.034 (0.012 to 0.056)**	0.011 (-0.010 to 0.032)
Psychosocial support status				
From friends and family				
Yes (ref. no)	0.193 (-0.145 to 0.530)	-0.164 (-0.465 to 0.137)	-0.026 (-0.314 to 0.263)	0.153 (-0.125 to 0.431)
From entertainment^$^				
Yes (ref. no)	0.211 (-0.136 to 0.559)	0.240 (-0.70 to 0.550)	-0.287 (-0.584 to 0.010)	0.475 (0.189 to 0.761)***
From other helping sources^##^				
Yes (ref. no)	-0.480 (-1.063 to 0.104)	-0.661 (-1.181 to -0.142)**	-0.220 (-0.719 to 0.278)	-0.506 (-0.987 to -0.026)**
Adjusted R-squared value	0.224	0.311	0.108	0.254

In addition, the study found that several factors negatively predicted the HRQoL score of psychological health. These included an increase in the age of the respondents (B = -0.029, p < 0.001), PSS score (B = -0.153, p < 0.001), age of the child (B = -0.098, p < 0.001), having a child with a neurodevelopmental disability other than the indexed child (B = -0.596, p < 0.05), time at diagnosis within two years (B = -0.337, p < 0.05) and support from helping sources other than friends and family (B = -0.661, p < 0.05).

Conversely, the study also found that a higher level of education (>grade X compared to grade X and below) (B = 0.410, p < 0.05), middle and upper SES (compared to lower SES) (B = 1.083, p < 0.001), and a higher coping score (B = 0.023, p < 0.05) were strong predictors of a high HRQoL score for psychological health.

The study also identified predictors of HRQoL scores for social relationships. The results indicated that having children with neurodevelopmental disabilities other than the indexed child (B = -0.518, p < 0.05) and a caregiver with a higher PSS score (B = -0.089, p < 0.001) were significant negative predictors of a low HRQoL score for social relationships. Conversely, the study found that middle and upper SES compared to lower SES (B = 0.541, p < 0.001), the indexed child being the first in the family (B = 0.331, p < 0.05), having married caregivers (B = 1.291, p < 0.001), and having a higher coping score (B = 0.034, p < 0.05) were significant positive predictors of a high HRQoL score for social relationships.

The study found that a low HRQoL for environmental health was significantly associated with a higher PSS score (B = -0.069, p < 0.001), older age of the indexed child (B = -0.055, p < 0.001), and psychosocial support from sources other than friends and family (B = -0.506, p < 0.05).

In contrast, caregivers who were mothers (B = 0.431, p < 0.05) had a higher level of education (>grade X compared to grade X and below) (B = 1.291, p < 0.001), and middle to upper SES (compared to those with a lower SES) (B = 1.320, p < 0.001), and having an entertainment source as psychosocial support (B=.475, P<.001) were significant positive predictors of a high HRQoL score for social relationships.

## Discussion

In recent times, the government of Bangladesh has taken several initiatives for the well-being of children with neurodevelopmental disabilities. However, information on children with developmental disabilities in Bangladesh is inadequate and often imprecise or underestimates the incidence. In Bangladesh, the percentage of children with disability ranges from less than 1.4% to 17.5% [13]. Children with developmental impairments seem to have complex health conditions and significant unmet health requirements that necessitate diagnosis and treatment. Having a child with a disability has a detrimental impact on parental life as it demands numerous adaptations for family members, as these children are entirely dependent on them.

Based on our findings, there was a significant negative relationship between the age of caregivers and the physical and psychological aspects of QoL, as well as a non-significant negative relationship with other domains. These results are consistent with previous research conducted on parents of children with neurodevelopmental disabilities in Bangladesh, which found that parents with higher QoL are related to higher age (r = 0.250, p < 0.01) [6]. Moreover, a study conducted among caregivers of children with special needs in Kelantan, Malaysia, also found that older age was directly associated with poorer QoL [14].

In addition, our findings revealed that the QoL of the caregiver decreased as the age of the child increased. This trend could be attributed to several factors, such as the worsening health condition of the child, the increasing need for nursing care, and the child’s physical and sexual maturation during puberty, which have been reported in previous studies [15]. These factors contribute to the increased burden experienced by caregivers who provide long-term care to a child with neurodevelopmental disabilities, leading to a decline in their QoL.

Our investigation explored how SES and education affect the QoL of parents having children with neurodevelopmental disabilities. Our results revealed a positive correlation between higher SES and education and better QoL, which is consistent with previous research conducted in Croatia on parents of children with intellectual disabilities [16], as well as other studies demonstrating that wealthier families have greater access to resources and can provide more support to their children [17]. Income and education are indicators of higher SES, linked to reduced emotional and physical stress and improved QoL.

Our study also found that mothers of children with neurodevelopmental disabilities reported lower physical QoL than fathers, which aligns with the findings of a survey conducted on parents of children with ASD conducted in Stockholm, Sweden [18]. Kazmi et al. [19] also discovered that mothers of children with disabilities in Khyber Pakhtunkhwa, Pakistan, had a lower QoL and were more susceptible to depression compared to fathers. Furthermore, our study revealed that mothers had lower QoL in other domains compared to fathers, possibly due to their increased responsibility in meeting the daily needs of their children with disabilities. Additionally, our study found that mothers reported worse mental health than fathers, which is consistent with the findings of Kheir et al. [20], who noted that mothers of children with disabilities had poor mental health and experienced increased levels of tiredness, fatigue, and bodily pain.

The marital status of parents having a child with neurodevelopmental disabilities was positively correlated with their QoL, which is in line with findings from Japan where 70% of parents of children with disabilities live together [21]. One possible explanation is that having a partner can emotionally strengthen caregivers by reducing loneliness and providing general support. In the context of Bangladesh, we observed that parents received psychosocial support from their spouse (77.6%), religious activities and communities (59.7%), and families (36.1%), as evidenced by higher mean scores in the social relationships’ domain compared to other domains, based on our findings.

Previous studies have reported that parents of children with disabilities need to pay for health services, which can result in financial crises for them [22]. It may indirectly lower the psychological QoL as it can create a feeling of guilt and pessimism in caregivers. Our study also found that parenting stress negatively affects the QoL in all four domains, which is consistent with the study findings of parents having children with intellectual disabilities [23]. The possible explanation is that rearing a child with neurodevelopmental disability is tiresome for parents as these types of children need continuous assistance in their daily activities. Parents may need to devote most of their time to caring for their impaired children, especially if the child has severe disabilities, leaving them unable to engage in other activities, limiting their social life, and negatively impacting their QoL.

Our study found that caregivers who were first-time parents of a child with neurodevelopmental disability and did not have any other children with neurodevelopmental disabilities had a higher QoL. This result was statistically significant, particularly in the social relationships domain of QoL, which is consistent with a study conducted in Dhaka city among parents of children with ASD [24]. The QoL of caregivers decreases with an increasing number of birth orders for children with neurodevelopmental disabilities. Although there is no evidence to support this theory, it can be explained by the fact that an increase in a child’s birth order results in a larger household with more children to care for, requiring caregivers to dedicate more time to raising them and reducing their QoL.

Our study found a significant association between occupational status and the QoL of caregivers of children with neurodevelopmental disabilities. Specifically, we found that unemployed caregivers had poorer QoL than those who were employed. This finding is consistent with a study conducted in Sweden, which also reported a link between unemployment and lower HRQoL [25]. The increased anxiety and despair among unemployed caregivers in our study could be due to the financial burden of caring for a child with a disability, which can be exacerbated by a lack of income.

Additionally, the sex of the child plays a vital role in the QoL of caregivers. Caregivers having a male child have better QoL. In the Bangladeshi context, having a male child is considered a blessing and a future asset of a family, although he has some impairments. According to a 2006 survey of 850 Bangladeshi families organized by Promoting Human Rights Education in Bangladesh, 93% of Bangladeshi families preferred a son, regarding him as a “blessing” to the family and society, while 93% saw girls as a “burden” [26] So, the sex of the child affects the QoL of caregivers to some extent. Having more than one child with neurodevelopmental disability negatively impacted parents’ QoL in all four domains, albeit only the psychological and social interactions category was determined to be statistically significant. Similar findings were reported in an Iranian study of parents with more than one child with special needs, where psychological QoL was shown to be poorer [27]. It can be stated that raising these children costs parents additional stress and mental harm.

In our study, coping strategies played a significant role in the QoL of parents having children with neurodevelopmental disabilities. It is believed that the ability of parents to utilize different coping mechanisms is a positive indicator that can lead to favorable mental health outcomes. Our study also found that a higher total coping score was significantly associated with the psychological and social relationships domain of QoL. This finding is consistent with a study conducted in India on caregivers of children with physical and mental disabilities [28].

In the cultural context of Bangladesh, there is a prevalent belief that disabilities are a result of bad parenting and a curse. Therefore, caregivers may feel ashamed of their child’s condition and how people perceive them. As a result, they use various types of distraction to cope with their stressful situations.

Social support plays a crucial role in helping parents of children with neurodevelopmental disabilities cope with stress, as it can provide them with emotional, practical, and informational assistance. Beresford [29] has also emphasized the importance of social support as a stress-moderating factor for parents of children with neurodevelopmental disabilities. In our study, we found that receiving psychosocial support from any source was statistically significant for improving psychological and environmental QoL. However, there was a negative correlation between receiving psychosocial support and QoL. While this may seem contradictory, it can be explained by the fact that individuals who seek assistance from other sources may have more severe or chronic problems, which can lead to poorer QoL outcomes. Additionally, seeking help from other sources may be linked to feelings of stigma or shame, which could further impact QoL negatively. Nevertheless, in a study by Hastings and Johnson [30], social support from family and friends was found to reduce stress levels among 141 UK parents who participated in intensive home-based behavioral interventions for their children with ASD.

Limitations of the study

Despite the efforts to investigate various aspects of the QoL of caregivers of children with neurodevelopmental disabilities, our study has certain limitations. The study used cross-sectional data to examine how parents of children with neurodevelopmental disabilities experienced stress, coping, psychosocial support status, and QoL. These factors are dynamic processes that may change over time. Future research should consider longitudinal studies to better understand how these factors evolve and interact throughout the caregiving journey. Moreover, the participants in our study were limited to caregivers who visited child development centers (SBK) at government medical college hospitals in Bangladesh, which may not represent the entire population of caregivers of children with neurodevelopmental disabilities in the country which may cause selection bias. Future studies should aim for a more diverse and inclusive sample that includes caregivers from various settings to improve the generalizability of the findings. Additionally, we could not analyze the degree and type of disability affecting the children in our study. Future research should strive to incorporate detailed assessments of the type and severity of the disability of the child to better understand how these factors influence caregiver stress, coping strategies, psychosocial support requirements, and overall QoL. This could lead to more tailored interventions that address the specific challenges faced by different groups of caregivers. By addressing these limitations, future research can provide a more comprehensive understanding of the factors influencing the QoL of caregivers and contribute to the development of targeted support systems.

## Conclusions

This study highlighting the challenges faced by caregivers of children with neurodevelopmental disabilities and their impact on the caregivers’ QoL found that factors such as the caregiver’s age, SES, education, and stress had a significant effect on the physical, psychological, and social aspects of their QoL. Coping strategies and psychosocial support were found to be significant predictors of QoL. The findings also suggest that caregivers experienced moderate levels of stress and had lower QoL scores, particularly in the physical and psychological health domains. The study suggests that to achieve the Sustainable Development Goals and improve the QoL of caregivers of children with neurodevelopmental disabilities in Bangladesh, it is crucial to provide parents of children with neurodevelopmental disabilities with adequate counseling to enhance their skills and empower them to advocate for child rights. As mothers are more susceptible in this regard, they should be given priority. Social support and various coping strategies should be developed to respond to the unique and changing needs of individuals and to alleviate parents’ stress related to raising a child with a disability. Further research should be conducted to measure the efficacy of the strategies implemented to enhance the QoL of caregivers of children with neurodevelopmental disabilities.
